# Influencing factors and early warning indicators for nonvertebral fractures in patients with Duchenne muscular dystrophy

**DOI:** 10.1093/jbmrpl/ziag098

**Published:** 2026-06-11

**Authors:** Changping Yan, Sen Kou, Shu Zhang, Pei Zhang, Yuandai Mao, Yang Liu, Shaoxia Wang, Ye Lin, Shanshan Kong, He Zhao, Sai Gao, Shengyuan Yu, Shiwen Wu

**Affiliations:** Department of Neurology, The First Medical Center of Chinese PLA General Hospital, Beijing 100853, China; School of Medicine, Nankai University, Tianjin 300071, China; Department of Neurology, The Third Medical Center of Chinese PLA General Hospital, Beijing 100039, China; Anhui Medical University, Hefei 230032, Anhui, China; Department of Neurology, The First Medical Center of Chinese PLA General Hospital, Beijing 100853, China; Center for Translational Genomics and Rare Diseases, The First Medical Center of Chinese PLA General Hospital, Beijing 100853, China; Beijing Key Laboratory of Gene Editing Therapy for Hereditary Neuromuscular Diseases, Beijing 100853, China; Department of Neurology, The First Medical Center of Chinese PLA General Hospital, Beijing 100853, China; Center for Translational Genomics and Rare Diseases, The First Medical Center of Chinese PLA General Hospital, Beijing 100853, China; Beijing Key Laboratory of Gene Editing Therapy for Hereditary Neuromuscular Diseases, Beijing 100853, China; Department of Neurology, The First Medical Center of Chinese PLA General Hospital, Beijing 100853, China; School of Medicine, Nankai University, Tianjin 300071, China; Department of Neurology, The First Medical Center of Chinese PLA General Hospital, Beijing 100853, China; Department of Neurology, The First Medical Center of Chinese PLA General Hospital, Beijing 100853, China; Center for Translational Genomics and Rare Diseases, The First Medical Center of Chinese PLA General Hospital, Beijing 100853, China; Beijing Key Laboratory of Gene Editing Therapy for Hereditary Neuromuscular Diseases, Beijing 100853, China; Department of Neurology, The First Medical Center of Chinese PLA General Hospital, Beijing 100853, China; Department of Neurology, The First Medical Center of Chinese PLA General Hospital, Beijing 100853, China; Department of Neurology, The First Medical Center of Chinese PLA General Hospital, Beijing 100853, China; Department of Neurology, The First Medical Center of Chinese PLA General Hospital, Beijing 100853, China; Department of Neurology, The First Medical Center of Chinese PLA General Hospital, Beijing 100853, China; School of Medicine, Nankai University, Tianjin 300071, China; Department of Neurology, The First Medical Center of Chinese PLA General Hospital, Beijing 100853, China; Center for Translational Genomics and Rare Diseases, The First Medical Center of Chinese PLA General Hospital, Beijing 100853, China; Beijing Key Laboratory of Gene Editing Therapy for Hereditary Neuromuscular Diseases, Beijing 100853, China

**Keywords:** Duchenne muscular dystrophy, nonvertebral fracture, ambulation, glucocorticoids, motor function

## Abstract

Nonvertebral fractures (NVFs) in patients with Duchenne muscular dystrophy (DMD) are often associated with an unfavorable prognosis. However, the influencing factors and early warning indicators for NVFs remain unclear. To address this knowledge gap, a retrospective cohort study was conducted across 2 centers involving 295 DMD patients who were evaluated between August 2013 and February 2022. Data collection included telephone callback records and outpatient face-to-face assessments. Among the 295 patients, 30 (10.2%) had experienced at least 1 NVF. The overall NVF incidence rate was 91.8 (95% CI, 62.4-130.3) per 10 000 person-years, representing a rate more than 5 times higher than that of traumatic NVFs in boys aged 0-14 yr. This finding underscores the significant burden of NVFs in DMD patients. Subsequent analysis investigated the association between the occurrence of the first NVF and various factors, including ambulant status, glucocorticoid (GC) regimen, and dystrophin isoform expression. Multivariate analysis revealed that ambulation was a significant risk factor for the first NVF, with an increased risk of 29.2-fold (95% CI, 3.9-217.3, *p <* .001). In contrast, a longer GC-naïve duration exhibited a protective effect against the first NVF, reducing the risk to 0.804-fold per year (95% CI, 0.691-0.936, *p =* .005). In patients aged 8-13 yr, motor function parameters 1 yr before the first NVF, including the 6-min walking distance (6MWD), the 10-m walking test time (10mWT), and the time from supine to standing (TSS), were significantly worse compared with those without NVF. Specifically, the 6MWD was shorter (*p* = .007), while 10mWT and TSS were longer (*p* = .025 and *p* = .020, respectively). Receiver operating characteristic curves indicated that a 6MWD of 248.2 m and a 10mWT of 9.85 s were the optimal cut-off values for predicting the onset of the first NVF, suggesting that a decline in the 6MWD to ~250 m and an increase in the 10mWT to ~10 s may serve as early warning indicators.

## Graphical Abstract

**Graphical Abstract:**
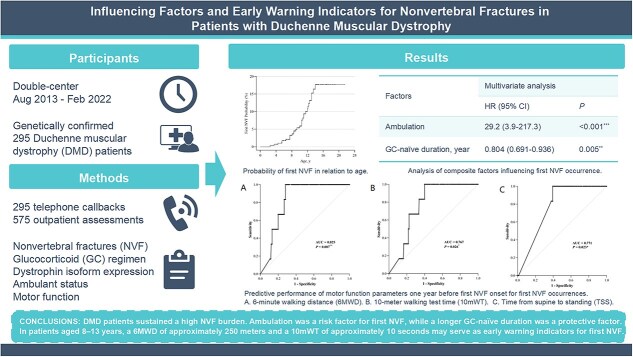

## Introduction

Duchenne muscular dystrophy (DMD) is a severe X-linked recessive muscle-wasting disease caused by mutations in the dystrophin gene, with an estimated prevalence of 1 in ~5000 live male neonates.^1^^,^^2^ The absence of dystrophin in the cytoskeleton of muscle cells leads to myofiber hypertrophy and necrosis, inflammation, fibrosis, and fat deposition in disorganized muscle tissue.^3^ Duchenne muscular dystrophy patients typically develop muscle weakness and hyperCKaemia at 3-5 yr of age, lose ambulation between 12 and 15 yr of age, and face premature death due to cardiorespiratory failure in their third or fourth decade of life.^4–7^

Bone health in DMD patients is compromised by both mechanical factors (eg, muscular degeneration, mechanical loading, and increased adiposity) and biochemical factors (eg, corticosteroid use, nutritional deficits, poor growth, and delayed puberty), leading to bone mass deficiency, bone geometry abnormalities, and decreased BMD. This results in reduced bone strength and increased fragility fractures.^8–11^ The incidence of long-bone fractures and symptomatic vertebral compression fractures in DMD patients is ~20%-30%, significantly higher than in healthy children.^10^

Nonvertebral fractures (NVFs), defined as all skeletal fractures excluding vertebral fractures (VFs), can cause significant pain, functional impairment, and a premature loss of ambulation. In patients with DMD, these injuries—predominantly involving the long bones—may even lead to life-threatening complications, such as fat embolism syndrome.^12^ Minimizing NVF occurrence is crucial for improving quality of life, guiding daily care, and prolonging survival. Unlike VFs, NVFs tend to occur after trauma, primarily affecting the extremities, resulting in different prognoses.^13^ However, previous studies have mainly focused on the incidence or location of NVFs; detailed evaluations of the associations among risk factors, early warning indicators, and the first NVF have been limited.^14–17^

To provide a basis for preventing NVFs and their complications, thereby enhancing the quality of life for DMD patients, this retrospective cohort study aimed to evaluate the associations between NVF incidence and potential risk factors, including glucocorticoid (GC) regimen status, ambulatory status, and dystrophin isoform expression. Additionally, the study compared the motor function parameters of DMD patients 1 yr before their first NVF onset (1-yr pre-NVF) with those of DMD patients without NVFs (non-NVF) from outpatient assessment records, identifying early warning indicators for NVF occurrence. This research aims to further explore these indicators to aid in the prevention of NVF-related complications and reduce the associated risks.

## Materials and methods

### Participants

A retrospective cohort study was conducted with a cohort of DMD patients attending the multidisciplinary team clinics at the Chinese People's Liberation Army (PLA) General Hospital First Medical Center and Third Medical Center from August 2013 to February 2022. A total of 320 outpatients fulfilled the following diagnostic criteria for DMD: (1) onset of weakness before 5 yr of age, (2) male sex, (3) proximal muscle weakness, (4) elevated serum creatine kinase, and (5) confirmation via DNA diagnosis. Data were collected through outpatient face-to-face assessments and telephone callbacks (both conducted in April 2022). After the callbacks, 25 patients were excluded due to 17 deaths and 8 refusals. Ultimately, 295 patients participated, with data obtained from 575 outpatient assessments and 295 telephone callbacks.

### Design

The mutation loci of the DMD gene for all 295 patients were recorded during their first outpatient review and initially tested using multiplex ligation-dependent probe amplification (MLPA) to detect deletions/duplications. Next-generation sequencing or Sanger sequencing was then applied to analyze MLPA-negative samples. Dystrophin isoform expression (Dp260, Dp140, Dp116, and Dp71) was determined based on DMD mutant genotyping according to Leiden Muscular Dystrophy pages (https://www.dmd.nl/).^18^^,^^19^ During each outpatient assessment, motor function parameters, including 6-min walking distance (6MWD), 10-m walking test time (10mWT), and time from supine to standing (TSS), were collected and measured by at least 2 neurologists. A 10mWT > 30 s or a TSS > 30 s was considered indicative of an inability to complete the test and was recorded as 30 s. Detailed information on NVF occurrence, ambulatory status, and GC regimens was obtained from all 575 outpatient assessments and 295 telephone callbacks. All NVFs were confirmed by clinical features and imaging, with the onset age (median if a range was reported) and location recorded. Nonambulant status was defined as a documented loss of independent ambulation, requiring the full-time use of assistive devices (electric or manual wheelchairs, scooters, etc.). For patients who had lost ambulation, the exact time of loss was recorded. GC regimens were documented as being adopted for patients who received prednisone (0.50-0.75 mg/kg/d) or deflazacort (0.90 mg/kg/d) for more than 3 consecutive months. Patients did not discontinue or change their GC regimens, and detailed documentation of the regimen and start time was maintained. The “GC-naïve duration” referred to the total period without GC treatment during the observation period. Elemental calcium (1200 mg) and vitamin D3 (250 IU) supplements were prescribed daily, with patients advised to obtain these nutrients through diet and sunlight exposure. No bisphosphonate or other bone antiresorptive or anabolic treatments were used in this cohort. All data were collected by trained outpatient clinicians from the Advanced Trial Outcome Measurement International Ltd team.

Out of the 575 outpatient assessments, data on the 6MWD, 10mWT, and TSS were recorded for 453 (78.8%), 565 (98.3%), and 560 (97.4%) assessments, respectively. To explore variations in motor functions, we identified a peak motor function around the age of 8 yr, with upward and downward trends before and after this age (Table S1). Records of 1-yr pre-NVF assessments (*n* = 8) ranged from the age of 6 to 13 yr, with only 2 records in the upward period (6-8 yr, too few for comparison) and 6 in the downward period (8-13 yr). Therefore, only records from patients aged 8-13 yr were analyzed, divided into the non-NVF (*n* = 250) and 1-yr pre-NVF (*n* = 6) groups. Other records (*n* = 319) were excluded. Among the 250 non-NVF assessments from patients aged 8-13 yr, data on the 6MWD, 10mWT, and TSS were recorded for 206 (82.4%), 248 (99.2%), and 243 (97.2%) assessments, respectively.

### Data analysis

Statistical analyses were performed using SPSS 28.0 (IBM Corporation, USA) and MedCalc Software version 20.2 (MedCalc Software Ltd, used for receiver operating characteristic [ROC] curve analysis). Categorical variables were presented as numbers or percentages, while continuous variables were presented as mean ± SD for normally distributed data or median (interquartile range) for non-normally distributed data. For the comparison of motor function parameters between groups, an independent-samples *t-*test was used for normally distributed variables, while the Mann-Whitney *U* test was applied for non-normally distributed variables. A 2-sided *p* < .05 was considered statistically significant.

Total person-years (PYs) were calculated as the sum of all observation years for all patients, with incidence rates presented per 10 000 PYs; 95% CIs were computed using the Byar method.

**Figure 1:**
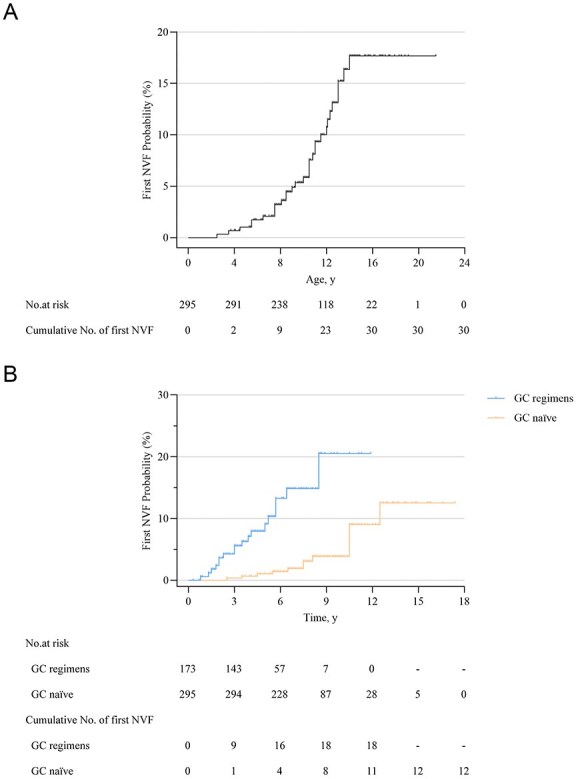
Probability of first nonvertebral fracture (NVF). (A) Cumulative probability of first NVF across all patients in relation to age. (B) Cumulative probability of first NVF in patients receiving glucocorticoid (GC) regimens (plotted against time on GC) compared with GC-naïve patients (plotted against GC-naïve duration). Patients who remained free of NVFs at the latest follow-up were included as censored cases (indicated by vertical lines on the Kaplan-Meier curve).

Kaplan-Meier curve was used to estimate the probability of the first NVF by age, with patients without NVFs censored. A univariate Cox proportional hazards model was employed to screen for potential risk or protective factors, including dystrophin isoform expression, ambulation, and the GC-naïve duration. A multivariate Cox proportional hazards model was then used to evaluate the impact of these factors on the time to the first NVF, expressed as hazard ratios (HR) with 95% CIs. Ambulation was entered into the Cox models as a time-varying covariate, reflecting each patient's ambulatory status over follow-up. Descriptive characteristics (Table 1) reflect each patient's status at the latest follow-up, whereas the Kaplan-Meier (Figure 1B) and incidence (Table 2) analyses treated GC status as time-varying, with each patient contributing GC-naïve time until GC initiation. In the Kaplan-Meier analysis, follow-up ended at the first NVF or at censoring for fracture-free patients; in the incidence analysis, each NVF was assigned to the GC regimens or GC-naïve group according to the patient's status at the time of NVF, with PYs apportioned accordingly.

Receiver operating characteristic curve was utilized to assess the predictive performance of motor function parameters 1-yr pre-NVF for the first NVF occurrences. The validity of the models was assessed using the area under the curve (AUC), and the optimal cut-off values were determined based on Youden’s index (J = Sensitivity + Specificity − 1).

## Results

### Clinical characteristics

In this cohort of 295 DMD patients, the median age at the latest follow-up was 11.3 (9.0, 13.8) years. As of the latest follow-up, 30 patients (10.2%) had experienced at least 1 NVF; 176 patients (59.7%) had been treated with GC—173 of whom had initiated GC before the end of their Kaplan-Meier follow-up (Figure 1A and B, respectively illustrate the probability of first NVF in relation to age for all patients and stratified by GC treatment status)—and 111 patients (37.6%) had lost ambulation. Regarding dystrophin isoform expression, Dp260, Dp140, Dp116, and Dp71 were detected in 73 (24.7%), 161 (54.6%), 281 (95.3%), and 289 (98.0%) patients, respectively (Table 1).

**Table 1:** Characteristics of DMD patients at the latest follow-up (*n* = 295).

**Individuals**	**NVF patients**	**Non-NVF patients**	**Total**
**GC treatment status**			
** GC regimens**	21 (11.9%)	155 (88.1%)	176
** GC-naïve**	9 (7.6%)	110 (92.4%)	119
**Ambulatory status**			
** Ambulant**	14 (7.6%)	170 (92.4%)	184
** Nonambulant**	16 (14.4%)	95 (85.6%)	111
**Dystrophin isoform**			
** Dp260**	8 (11.0%)	65 (89.0%)	73
** Dp140**	15 (9.3%)	146 (90.7%)	161
** Dp116**	27 (9.6%)	254 (90.4%)	281
** Dp71**	28 (9.7%)	261 (90.3%)	289

Abbreviations: DMD, Duchenne muscular dystrophy; GC, glucocorticoid; NVF, nonvertebral fracture.

### NVF incidence and locations

A total of 31 NVFs were recorded in the 30 NVF patients, 1 of whom experienced 2 NVFs. Over a total observation time of 3376.7 PYs, the overall NVF incidence rate was 91.8 per 10 000 PYs (95% CI, 62.4-130.3). The incidences of NVFs by age and GC treatment status are presented in detail in Table 2.

Thirty-five body parts were affected by NVFs, including 4 instances of concurrent double-site fractures. Specifically, there were 11 upper-extremity fractures (31.4%), 22 lower-extremity fractures (62.9%), and 2 other fractures (5.7%). The specific locations of the NVFs are listed in Table 3. Lower-extremity fractures often result in a poor prognosis. Among the 19 patients who experienced lower-extremity fractures, 10 (52.6%) lost ambulation within 1 yr.

**Table 2:** Incidence of NVF by age groups and GC treatment status.

**NVF characteristics**	**Number of NVFs**	**NVF incidence per 10 000 PYs (95% CI)**
**Age (yr)**		
**0-4.9**	3	20.4 (4.1-59.6)
**5.0-9.9**	11	86.2 (43.0-154.3)
**10.0-14.9**	17	302.3 (176.0-484.0)
**≥15.0**	0	0
**GC treatment status**		
**GC regimens**	19	203.2 (122.3-343.4)
**GC-naïve**	12	49.1 (25.4-85.9)
**Total**	31	91.8 (62.4-130.3)

Abbreviations: GC, glucocorticoid; NVF, nonvertebral fracture; PY, person-year.

**Table 3:** Specific body locations of NVF occurrences.

**Location**	**Number of NVF sites**		
	**GC-naïve**	**GC regimens**	**Total**
**Upper extremities**	4	7	11
**Elbow**	1	0	1
** Humerus**	0	3	3
** Ulna**	1	2	3
** Radius**	1	2	3
** Wrist**	1	0	1
**Lower extremities**	8	14	22
** Femur**	2	2	4
** Tibia**	1	2	3
** Fibula**	3	2	5
** Ankle**	1	5	6
** Metatarsal**	1	2	3
** Knee**	0	1	1
**Other locations**	2	0	2
** Clavicle**	1	0	1
** Nasal bone**	1	0	1
**Total**	14	21	35

Abbreviations: GC, glucocorticoid; NVF, nonvertebral fracture.

### Multivariate analysis for correlates of first NVF

Univariate analysis revealed that ambulation was associated with an increased risk of the first NVF, whereas a longer GC-naïve duration was associated with a decreased risk. Dystrophin isoforms, however, did not significantly influence the risk. Multivariate analysis confirmed that ambulation and the GC-naïve duration remained significant predictors of the first NVF. Patients who retained ambulation had a 29.2-fold increased risk (HR = 29.2, 95% CI, 3.9-217.3, *p* < .001), while each additional year of GC-naïve status reduced the risk to 0.804-fold (HR = 0.804, 95% CI, 0.691-0.936, *p* = .005) (Table 4).

**Table 4:** Analysis of composite factors influencing the occurrence of first NVF.

**Factors**	**Univariate analysis**			**Multivariate analysis**		
	***β***	**HR (95% CI)**	***p***	***β***	**HR (95% CI)**	***p***
**Ambulation**	3.462	31.880 (4.302-236.245)	<.001^b^	3.375	29.236 (3.933-217.327)	<.001
**Dystrophin isoform**						
** Dp260**	0.055	1.057 (0.470-2.374)	.894			
** Dp140**	−0.187	0.829 (0.405-1.697)	.608			
** Dp116**	−0.977	0.377 (0.114-1.243)	.109			
** Dp71**	−1.332	0.264 (0.063-1.111)	.069			
**GC-naïve duration, yr**	−0.273	0.761 (0.644-0.900)	.001^a^	−0.218	0.804 (0.691–0.936)	.005^a^

a *p* < .01.

b *p* < .001.

Abbreviations: β, regression coefficient; GC, glucocorticoid; HR, hazard ratio; NVF, nonvertebral fracture.

### Group analysis for early warning factors

**Figure 2:**
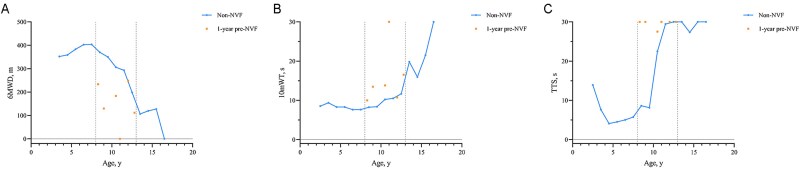
Median motor function parameters in outpatient assessments for patients aged 8-13 yr. (A) 6-min walking test distance (6MWD). (B) 10-m walking test time (10mWT). (C) Time from supine to standing (TSS). The motor function parameters of patients assessed 1 yr before their first nonvertebral fracture (NVF) onset (1-yr pre-NVF) are indicated by individual data points, while the median motor function parameters of non-NVF patients by age are indicated by the connecting lines. In the 10mWT and TSS, durations exceeding 30 s are capped at 30 s for presentation.

In outpatient assessments of patients aged 8-13 yr, no significant difference in age was observed between the 1-yr pre-NVF and non-NVF groups. However, the 10mWT and TSS were significantly longer in the 1-yr pre-NVF group compared with the non-NVF group (*p* = .025 and *p* = .020, respectively), while the 6MWD was significantly shorter (*p* = .007) (Table 5). Figure 2 visually illustrates the decline in motor function in the 1-yr pre-NVF group compared with the non-NVF group.

**Table 5:** Comparison of motor function parameters between 1-yr pre-NVF patients and non-NVF patients.

**Group**	**1-yr pre-NVF**	**Non-NVF**	***p***
**10mWT** ^**a**^			
** No.**	6	248	-
** Age (yr)**	10.75 (8.83, 12.20)	9.70 (8.80, 10.80)	.248
** Median (s)**	13.64 (10.55, 19.91)	9.09 (7.56, 12.57)	.025^b^
**6MWD**			
** No.**	6	206	-
** Age (yr)**	10.75 (8.83, 12.20)	9.80 (8.80, 11.00)	.294
** Median (m)**	156.5 (83.8, 237.0)	340.0 (216.9, 400.5)	.007^c^
**TSS** ^**a**^			
** No.**	6	243	-
** Age (yr)**	10.75 (8.83, 12.20)	9.70 (8.80, 10.80)	.251
** Median (s)**	>30 (29.38, >30)	13.16 (6.54, >30)	.020^b^

a The 10mWT and TSS over 30 s were recorded as >30.

b *p* < .05.

c *p* < .01.

Abbreviations: 1-yr pre-NVF, 1 yr before first NVF onset; 10mWT, 10-m walking test time; 6MWD, 6-min walking distance; NVF, nonvertebral fracture; TSS, time from supine to standing.

**Figure 3:**
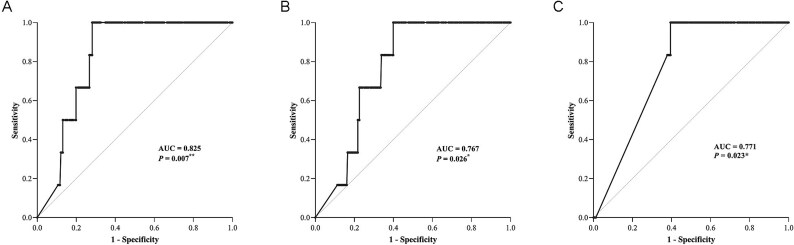
Predictive performance of motor function parameters 1 yr before first nonvertebral fracture (NVF) onset (1-yr pre-NVF) for first NVF occurrences in patients aged 8-13 yr. (A) 6-min walking test distance (6MWD). (B) 10-m walking test time (10mWT). (C) Time from supine to standing (TSS). AUC: area under the curve. ^*^p < .05; ^**^p < .01.

To identify early warning indicators for NVF in patients aged 8-13 yr, ROC curves were constructed using the 10mWT, 6MWD, and TSS data from the 1-yr pre-NVF and non-NVF groups. The AUC for the 10mWT was 0.767 (*p* = .026), with a cut-off value of 9.85 s (sensitivity 100.00%, specificity 60.08%, *J* = 0.6008). The AUC for the 6MWD was 0.825 (*p* = .007), with a cut-off value of 248.2 m (sensitivity 100.00%, specificity 71.84%, *J* = 0.7184). The AUC for the TSS was 0.771 (*p* = .023), with a cut-off value of 26.91 s (sensitivity 100.00%, specificity 60.49%, *J* = 0.6049) (Figure 3A-C).

## Discussion

To our knowledge, this is the first study to systematically analyze the associations between the incidence of the first NVF and potential risk factors, as well as to explore early warning indicators for first NVF occurrence. Our results showed that ambulation was an independent risk factor for the first NVF; while a prolonged GC-naïve duration served as a protective factor. In DMD patients aged 8-13 yr, motor function parameters were significantly worse 1 yr before the first NVF. Specifically, a 6MWD of 248.2 m and a 10mWT of 9.85 s were determined to be the optimal cut-off values for predicting the onset of the first NVF.

In this cohort, the overall NVF incidence rate was 91.8 per 10 000 PYs, peaking at 302.3 per 10 000 PYs in the age range of 10.0-14.9 yr. According to a 2014 census of traumatic fractures in China, the incidence of traumatic fractures in boys aged 0-14 yr was ~17.5 per 10 000 PYs, of which NVFs accounted for about 16.8 per 10 000 PYs.^20^ Therefore, the NVF incidence in our cohort was more than 5 times higher than that in their peers. Of the patients in our study, 10.2% experienced at least 1 NVF, which is comparable with the 13.5% reported in a recent UK NorthStar database study but lower than the 20%-40% reported in some previous studies.^9^^,^^14^^,^^15^^,^^21–23^ This discrepancy might be attributable to the fracture prevention advice provided during follow-up visits, which is based on general guidance for DMD patients and their families.^24^

The use of GC and the negative effects of dystrophic muscle on bone contribute to a high fracture rate, particularly in the lower extremities.^25^^,^^26^ Lower-extremity long-bone fractures often lead to the permanent loss of independent ambulation in DMD patients. Consistent with previous studies, our data showed that about half of the patients lost ambulation within 1 yr after the first lower-extremity NVF.^27^^,^^28^ The goal for ambulant DMD patients with lower-extremity fractures is to initiate weight-bearing training as soon as possible; however, more than half of the patients did not adhere to this program, thereby contributing to the loss of ambulation.

Glucocorticoids exert a strong anti-inflammatory effect by reducing the inflammatory response in dystrophin-deficient muscle, thereby preserving muscle strength and prolonging independent ambulation, as well as maintaining cardiac and respiratory function.^28–30^ However, long-term GC therapy can cause substantial bone mineral loss, deterioration of cortical and cancellous bone strength, leading to osteoporosis and an increased fracture risk.^31^ GC therapy acts as a “double-edged sword”: while it delays the loss of ambulation—thereby sustaining beneficial mechanical loading on bone—the cumulative detrimental impact of GC exposure on bone fragility may outweigh this mechanical benefit. A longer GC-naïve duration likely preserves native bone microarchitecture and mineral density prior to the onset of GC-induced skeletal deterioration, thereby narrowing the “window of vulnerability,” during which patients face both heightened drug-induced bone fragility and an increased risk of falls from progressive muscle weakness. Additionally, GC regimens can cause obesity, which increases exercise burden, reduces flexibility, decreases vitamin D concentration due to its adipocyte deposition, increases leptin-producing adipocytes that harm bone health, and decreases mobility due to intramuscular fat replacement.^32–35^ A retrospective study revealed that obesity puts DMD patients at risk of fractures at a younger age.^36^ In summary, selecting the optimal time to initiate GC regimens for DMD patients is imperative.

Prednisone and deflazacort are the most commonly used GCs. Recent randomized clinical trials showed no significant difference in therapeutic effect between daily prednisone (0.75 mg/kg) and daily deflazacort (0.90 mg/kg).^37–40^ Deflazacort has been widely adopted in clinical practice due to its reportedly lower incidence of side effects, such as obesity, a cushingoid appearance, behavioral changes, and gastrointestinal symptoms.^41^^,^^42^ Regarding the destructive effect of deflazacort on bones compared with other hormones, various studies have not yet reached a consensus. Previous experimental studies have shown that, compared with dexamethasone and cortisol, deflazacort may have a bone-sparing effect by affecting bone formation. However, clinical studies have shown an increase in fracture frequency with deflazacort.^21^^,^^43^^,^^44^ In our cohort, despite the small number of patients treated daily with deflazacort (*n* = 14), we observed a trend toward a higher NVF incidence compared with those receiving daily prednisone (*n* = 162): 626.6 (95% CI, 201.9-1462.0) per 10 000 PYs vs 163.7 (95% CI, 89.4-274.7) per 10 000 PYs. Specialists should carefully consider the indications for deflazacort in treating DMD.

We identified a 29.2-fold increased risk of the first NVF in ambulant DMD patients, suggesting that the incidence of long-bone fractures increases while DMD patients remain ambulant. Similar to our findings, Joseph et al. also estimated that the hazard for the first fracture (both VF and NVF) was increased by 3.7-fold in ambulant patients.^21^^,^^45^ Trauma is the most common cause of fractures in the general population, and NVFs account for nearly 97% of traumatic fractures in Chinese boys aged 0-14 yr.^20^ Ambulant DMD patients typically fall within the 0-14-yr age window, a period characterized by rapid skeletal growth. During this phase, bone expansion often outpaces mineralization, creating a physiological window of transient cortical fragility.^46^ Concurrently, these patients engage in physical activities typical of childhood; however, the combination of frequent movement and disease-related muscle weakness disproportionately elevates the risk of falls and collisions. Conversely, the transition to a non-ambulant status drastically curtails trauma exposure. Consequently, the observed association between ambulation and NVF risk should not be interpreted as ambulation itself being inherently harmful, but rather as reflecting the combined influence of retained mobility, greater activity-related trauma exposure, and age-related skeletal characteristics.

Although GCs prolong the independent ambulatory phase by reducing muscle degeneration, they also introduce a non-negligible risk of NVF.^47^ However, ambulation remains beneficial for bone health, as immobilization and lack of mechanical stimulation can lead to decreased BMD and osteoporosis.^48^^,^^49^ Duchenne muscular dystrophy patients are encouraged to engage in gentle exercises, such as swimming, cycling, and low-resistance strength training, to enhance muscle function while minimizing the risk of traumatic fractures.^50^ Additionally, guardians should ensure that DMD patients are protected from potential trauma during ambulation.

Previous studies have shown that DMD patients typically lose ambulation by age 13, with peak motor function reached around age 7-8.^7^^,^^47^^,^^51^ Among the DMD patients aged 8-13 yr in our cohort, significantly worse motor function was observed in the 1-yr pre-NVF patients compared with the non-NVF patients, as indicated by a shorter 6MWD, a longer 10mWT, and a longer TSS. Moreover, the cut-off values for predicting the onset of the first NVF were 248.2 m for the 6MWD, 9.85 s for the 10mWT, and 26.91 s for the TSS. However, based on the shape of the ROC curves, we believe that it is more reasonable to use a 6MWD of 248.2 m and a 10mWT of 9.85 s as early warning indicators for the first NVF in DMD patients aged 8-13 yr (Figure 3A-C). Therefore, a 6MWD of ~250 m and a 10mWT of ~10 s suggest poor motor function, an increased risk of trauma, and susceptibility to NVF.

There are several limitations to our study. First, data were not collected from fixed or predefined interval assessments for all patients, potentially leading to an underestimation of the NVF incidence. Second, although we identified ambulation as a strong risk factor for the first NVF, residual confounding by age and activity level cannot be fully excluded, as the ambulant phase inherently overlaps with the period of rapid skeletal growth and heightened physical activity in DMD children. Third, the impact of other interventions on bone development, such as vitamin D supplementation and weight training, was not quantitatively assessed. Fourth, the ROC-derived thresholds for 6MWD (248.2 m) and 10mWT (9.85 s) were based on a small subset of pre-NVF cases (*n* = 6) within the decline phase (aged 8-13 yr). Given the limited sample size, these values should be regarded as exploratory early warning indicators rather than established clinical thresholds. Fifth, the inclusion of participants from 2 centers in China may limit the generalizability of our findings to populations with different cultural backgrounds and healthcare systems, particularly with respect to the specific numerical thresholds identified for the 6MWD and 10mWT. Future large-scale, multicenter, and multi-ethnic cohort studies are warranted to validate these exploratory findings.

In conclusion, the incidence of NVFs in DMD patients was considerably higher than that in their peers, highlighting a substantial burden of NVFs in DMD patients. Multivariate analysis revealed that ambulation was a powerful risk factor for NVFs, while a prolonged GC-naïve duration was a protective factor. In patients aged 8-13 yr, motor function parameters may serve as early warning indicators for the first NVF. When a 6MWD declines to ~250 m and a 10mWT increases to ~10 s, patients should be advised to implement preventive measures to reduce the risk of NVFs.

## Supplementary Material

Supplementary_Table_1_ziag098

## Data Availability

The anonymized dataset is available from the corresponding authors upon reasonable request.
