# Supplementary material for: Influencing factors and early warning indicators for nonvertebral fractures in patients with Duchenne muscular dystrophy
Source: JBMR Plus. 2026 Jun 11;10(8):ziag098. doi: 10.1093/jbmrpl/ziag098 (PMC13332812; doi:10.1093/jbmrpl/ziag098)
Supplement: Supplementary_Table_1_ziag098 [file supplementary_table_1_ziag098.docx]

**Supplementary Table 1. Motor Function Parameters of Non-NVF Patients Stratified by Age Groups**

| **Age, y** | **10mWT^a^, s** | **6MWD, m** | **TSS^a^, s** |
| --- | --- | --- | --- |
| 2.0-2.9 | 8.53 (8.53, 8.53) | - | 13.90 (13.90, 13.90) |
| 3.0-3.9 | 9.38 (8.69, 11.11) | 352.0 (326.4, 353.9) | 7.65 (6.00, 9.97) |
| 4.0-4.9 | 8.31 (6.80, 9.10) | 358.9 (343.3, 414.0) | 4.09 (2.90, 5.06) |
| 5.0-5.9 | 8.30 (7.30, 9.37) | 383.0 (336.7, 411.0) | 4.51 (3.72, 5.99) |
| 6.0-6.9 | 7.63 (6.87, 8.46) | 402.8 (350.0, 432.0) | 5.02 (3.77, 7.15) |
| 7.0-7.9 | 7.62 (6.83, 8.51) | 403.6 (354.8, 444.9) | 5.73 (3.92, 9.28) |
| 8.0-8.9 | 8.24 (7.17, 9.65) | 370.1 (327.1, 409.2) | 8.61 (5.13, 26.72) |
| 9.0-9.9 | 8.38 (7.21, 11.42) | 350.0 (275.0, 414.1) | 8.13 (5.90, >30) |
| 10.0-10.9 | 10.24 (7.87, 14.44) | 306.0 (187.8, 407.0) | 22.50 (7.09, >30) |
| 11.0-11.9 | 10.50 (8.56, 21.65) | 293.0 (142.7, 367.3) | 29.45 (9.58, >30) |
| 12.0-12.9 | 11.67 (10.04, 19.27) | 198.4 (140.4, 285.5) | >30 (>30, >30) |
| 13.0-13.9 | 19.83 (17.98, 27.68) | 106.5 (78.8, 131.0) | >30 (>30, >30) |
| 14.0-14.9 | 15.96 (13.91, 18.47) | 119.4 (97.5, 207.7) | 27.35 (16.18, >30) |
| 15.0-15.9 | 21.49 (17.23, 25,74) | 127.4 (63.7, 191.1) | >30 (>30, >30) |
| 16.0-16.9 | >30 (>30, >30) | 0 (0,0) | >30 (>30, >30) |

^a^: The 10mWT and TSS over 30 seconds were recorded as >30. Abbreviations: 10mWT: 10-meter walking test time; 6MWD: 6-minute walking distance; m: meter; s: second; TSS: time from supine to standing; y: year.
